# Professional Uncertainty and Disempowerment Responding to Ethnic Diversity in Health Care: A Qualitative Study

**DOI:** 10.1371/journal.pmed.0040323

**Published:** 2007-11-13

**Authors:** Joe Kai, Jackie Beavan, Christina Faull, Lynne Dodson, Paramjit Gill, Angela Beighton

**Affiliations:** 1 Division of Primary Care, University of Nottingham Graduate Medical School, United Kingdom; 2 LOROS, Leicester, United Kingdom; 3 United Hospitals Birmingham National Health Service Trust, Birmingham, United Kingdom; 4 Department of General Practice and Primary Care, University of Birmingham, United Kingdom; University of California Davis, United States of America

## Abstract

**Background:**

While ethnic disparities in health and health care are increasing, evidence on how to enhance quality of care and reduce inequalities remains limited. Despite growth in the scope and application of guidelines on “cultural competence,” remarkably little is known about how practising health professionals experience and perceive their work with patients from diverse ethnic communities. Using cancer care as a clinical context, we aimed to explore this with a range of health professionals to inform interventions to enhance quality of care.

**Methods and Findings:**

We conducted a qualitative study involving 18 focus groups with a purposeful sample of 106 health professionals of differing disciplines, in primary and secondary care settings, working with patient populations of varying ethnic diversity in the Midlands of the UK. Data were analysed by constant comparison and we undertook processes for validation of analysis. We found that, as they sought to offer appropriate care, health professionals wrestled with considerable uncertainty and apprehension in responding to the needs of patients of ethnicities different from their own. They emphasised their perceived ignorance about cultural difference and were anxious about being culturally inappropriate, causing affront, or appearing discriminatory or racist. Professionals' ability to think and act flexibly or creatively faltered. Although trying to do their best, professionals' uncertainty was disempowering, creating a disabling hesitancy and inertia in their practice. Most professionals sought and applied a knowledge-based cultural expertise approach to patients, though some identified the risk of engendering stereotypical expectations of patients. Professionals' uncertainty and disempowerment had the potential to perpetuate each other, to the detriment of patient care.

**Conclusions:**

This study suggests potential mechanisms by which health professionals may inadvertently contribute to ethnic disparities in health care. It identifies critical opportunities to empower health professionals to respond more effectively. Interventions should help professionals acknowledge their uncertainty and its potential to create inertia in their practice. A shift away from a cultural expertise model toward a greater focus on each patient as an individual may help.

## Abstract 2

From a qualitative study, Joe Kai and colleagues have identified opportunities to empower health professionals to respond more effectively to challenges in their work with patients from diverse ethnic communities.

## Editors' Summary

**Background.:**

Communities are increasingly diverse in terms of ethnicity (belonging to a group of people defined by social characteristics such as cultural tradition or national origin) and race (belonging to a group identified by inherited physical characteristics). Although health professionals and governments are striving to ensure that everybody has the same access to health care, there is increasing evidence of ethnic inequalities in health-care outcomes. Some of these inequalities reflect intrinsic differences between groups of people—Ashkenazi Jews, for example, often carry an altered gene that increases their chance of developing aggressive breast cancer. Often, however, these differences reflect inequalities in the health care received by different ethnic groups. To improve this situation, “cultural competence” has been promoted over recent years. Cultural competence is the development of skills by individuals and organizations that allow them to work effectively with people from different cultures. Health professionals are now taught about ethnic differences in health beliefs and practices, religion, and communication styles to help them provide the best service to all their patients.

**Why Was This Study Done?:**

Numerous guidelines aim to improve cultural competency but little is known about how health professionals experience and perceive their work with patients from diverse ethnic groups. Is their behavior influenced by ethnicity in ways that might contribute to health care disparities? For example, do doctors sometimes avoid medical examinations for fear of causing offence because of cultural differences? If more were known about how health professionals handle ethnic diversity (a term used here to include both ethnicity and race) it might be possible to reduce ethnic inequalities in health care. In this qualitative study, the researchers have explored how health professionals involved in cancer care are affected by working with ethnically diverse patients. A qualitative study is one that collects nonquantitative data such as how doctors “feel” about treating people of different ethnic backgrounds; a quantitative study might compare clinical outcomes in different ethnic groups.

**What Did the Researchers Do and Find?:**

The researchers enrolled 106 doctors, nurses, and other health-related professionals from different health-service settings in the Midlands, an ethnically diverse region of the UK. They organized 18 focus groups in which the health professionals described their experiences of caring for people from ethnic minority backgrounds. The participants were encouraged to recall actual cases and to identify what they saw as problems and strengths in their interactions with these patients. The researchers found that the health professionals wrestled with many challenges when providing health care for patients from diverse ethnic backgrounds. These challenges included problems with language and with general communication (for example, deciding when it was acceptable to touch a patient to show empathy). Health professionals also worried they did not know enough about cultural differences. As a result, they said they often felt uncertain of their ability to avoid causing affront or appearing racist. This uncertainty, the researchers report, disempowered the health professionals, sometimes making them hesitate or fail to do what was best for their patient.

**What Do These Findings Mean?:**

These findings reveal that health professionals often experience considerable uncertainty when caring for ethnically diverse patients, even after training in cultural competency. They also show that this uncertainty can lead to hesitancy and inertia, which might contribute to ethnic health care inequalities. Because the study participants were probably already interested in ethnic diversity and health care, interviews with other health professionals (and investigations of patient experiences) are needed to confirm these findings. Nevertheless, the researchers suggest several interventions that might reduce health care inequalities caused by ethnic diversity. For example, health professionals should be encouraged to recognize their uncertainty and should have access to more information and training about ethnic differences. In addition, there should be a shift in emphasis away from relying on knowledge-based cultural information towards taking an “ethnographic” approach. In other words, health professionals should be helped to feel able to ask their patients about what matters most to them as individuals about their illness and treatment.

**Additional Information.:**

Please access these Web sites via the online version of this summary at http://dx.doi.org/10.1371/journal.pmed.0040323.

- Information on cultural competence and health care is available from the US National Center for Cultural Competence (in English and Spanish) and DiversityRx
- PROCEED (Professionals Responding to Cancer in Ethnic Diversity) is a multimedia training tool for educators within the health and allied professions developed from the results of this study; a press release on PROCEED is available from the University of Nottingham
- Transcultural Health Care Practice: An educational resource for nurses and health care practitioners is available on the web site of the UK Royal College of Nursing

## Introduction

Health care occurs within contexts of increasing community diversity. Nearly one in ten of the UK, and over one in four of the US populations identify themselves as from an ethnic minority [1,2]. Concerns about ethnic disparities in health and health care have long preoccupied communities, policy makers, and health care providers alike [3–5]. Meanwhile, evidence of ethnic inequalities in access to care, health care received, and health care outcomes is increasingly exposed [5–10].

Policy commitment to reducing such inequality has been strengthened by the Race Relations (Amendment) Act 2000 in the UK, which requires all statutory services to not only avoid discrimination but to actively promote and report on equality in service delivery [11]. In the US, goals for improving minority health are a key part of the Healthy People 2010 initiative [12].

Recommendations for health system interventions include more systematic collection and use of ethnicity data; improvements in provision of interpreting services, culturally appropriate information, and lay community health workers; alongside enhanced patient education and better health professional education and training [5,13–17]. Despite policy initiatives and legislation, robust evidence informing strategies to enhance quality of care and reduce inequalities in day-to-day health care practice remains limited [18]. Here, the need for “cultural competence” has been promoted at the system level and within the patient–health professional encounter [16]. The latter typically refers to a set of knowledge and skills in which health professionals might be trained in order to better respond to patient diversity [19].

It has long been recognised that cultural diversity matters in clinical practice, shaping beliefs, values, and behaviours, and influencing care [20,21]. There are now many examples of guidelines and models for “culturally competent” health care [22–26], which generally underscore developing awareness of personal biases, values, and assumptions. The cultural competence model advocated in most guidelines entails gaining knowledge of cultural differences, for example in health beliefs and practices, religion, and communication styles. This system sometimes incorporates approaches to eliciting patient's beliefs and understanding about symptoms and illness [20].

Despite this movement to improve intercultural care, surprisingly little is known about how practising health professionals themselves experience and perceive their work with patients in ethnically diverse contexts. There is a lack of relevant qualitative data, in particular concerning hypotheses that health professional behaviour may be influenced by patient ethnicity in ways that may contribute to disparities [27]. This study formed part of a wider initiative to support care of people with cancer from ethnic minorities [28], where there is growing evidence of inequality in care and outcomes [29].

Our enquiry focuses on “ethnic” diversity to embrace ethnicity and “race” while recognizing other aspects of social diversity; for example, socioeconomic status, gender, or age may exert cultural influences on health care encounters. We refer to ethnicity as the social concept of ethnic identity—people may identify themselves as belonging to social groupings because they differ culturally in ways such as language, religions, food, lifestyle, or geographical origin or differ in physical features as in “race.” Using cancer care as a clinical context, we aimed to explore how practising health professionals experience and perceive their work with patients of ethnicity different from their own to inform interventions to enhance quality of care.

## Methods

### Sampling and Ethical Approval

Participants were sampled purposefully from a range of health service settings and networks in the Midlands of the UK (primary care, hospitals, workforce confederations, and clinical care networks) to include health professionals of varying disciplines and experiences in working with ethnically diverse patients in a variety of care settings.

The study protocol, participant information, and consent procedures were reviewed by a UK multi-centre research ethics committee, which had no ethical objections.

### Data Generation

We used focus groups rather than one-on-one interviews to seek insights into attitudes, opinions, and underlying assumptions that group interactions can enable, allowing participants to discuss each others' perspectives [30]. Following invitations sent via local service contacts, professionals willing to participate were selected so that each focus group was either generally homogenous by discipline to promote sharing of experiences and equality of professional power (13 groups), or multidisciplinary to encourage exploration of views from members of the same care team (five groups).

Following a pilot focus group to develop our initial topic guide and procedures, 18 focus groups (range 5–11 participants) involving 106 respondents were facilitated by JB, with other authors cofacilitating some groups, mostly in participants' work settings. This large number of interviews enabled generation of data, and their saturation, from a wide range of health professionals and settings (Table 1). Interviews commenced with a broad introductory question, “Could you comment on any experiences you have had when caring for people from an ethnic minority background?”. Participants' recall of actual cases was then used to generate discussion of their experiences, perceptions, perceived strengths, and concerns in health care interaction with patients from minority ethnic communities. Group discussions lasted between one and a half to two hours, and were audiotaped and transcribed verbatim.

**Table 1:**
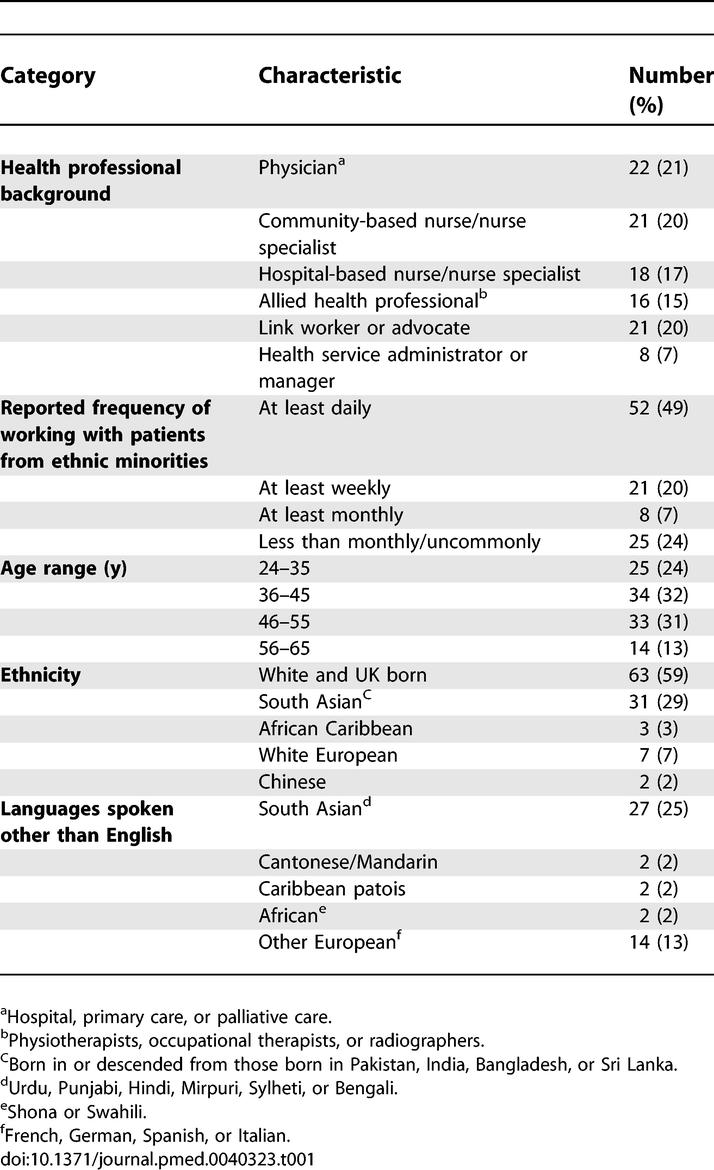
Characteristics of Respondents (*n* = 106)

The characteristics of participants (74 women, 32 men) and their range of experience of working with patients from ethnic “minorities” are shown in Table 1.

### Data Analysis and Validation

We used constant comparison, in which data were collected and analysed concurrently [31], enabling emergent themes and ideas to be incorporated and explored in subsequent interviews, to develop categories. Coding, assisted by N-Vivo software, was developed and discussed between JK (clinical primary care academic), JB (research fellow with linguistics and education background), and CF (consultant in palliative care medicine). New data were used and deviant cases [31] sought to assess the integrity of the categories identified, with data generation continuing until no new categories were emerging, suggesting saturation [31].

Preliminary findings were fed back to and discussed with a group of seven health care advocates from minority ethnic communities working with patients, and an eight-strong multidisciplinary advisory group with health service and academic expertise in cultural diversity. Focus group participants were sent, and invited to comment on, a summary of results, and seven also attended a further focus group facilitated by JB and JK to discuss and check validity of our interpretation of the data [32]. These processes confirmed and helped further refine analysis.

## Results

While respondents sought to provide professional standards of care for patients from diverse ethnic backgrounds, they wrestled with a range of challenges. These challenges included communication, language, and working with patients in the context of their families. The results presented here focus on professionals' common experience of uncertainty and the disempowering effects this uncertainty had on professionals' clinical practice. These experiences occurred in the context of professionals' emphasis on cultural difference and knowledge-based cultural expertise.

### Professional Uncertainty

Professionals' uncertainty about how to negotiate encounters involving patients with perceived cultural differences to themselves featured across accounts and our ethnically diverse sample. Faced with the apparently unfamiliar, health professionals could feel apprehensive and uncomfortable. They struggled to reconcile the desire to act appropriately with the perception that cultural barriers between themselves and patients could be insurmountable. These barriers included the practical exigencies of lacking a shared language with some patients and mediation of communication by others. However, uncertainty about differing cultural beliefs, practices, and attitudes of patients in relation to illness and care remained. Respondents emphasised how they lacked cultural knowledge and understanding to address patients' needs. They worried about the compromising effects this deficiency might have for them or their patients were they to “make a mistake.”

Respondents' uncertainty was unsettling to them emotionally and personally. Their uncertainty was most often driven by a fear of doing the wrong thing. They wanted to be “culturally appropriate” in their interactions with patients or their families so as not to cause offence (Box 1).

Some professionals worried that their lack of specific cultural knowledge may appear discriminatory or racist, creating further uncertainty about how to act. This was expressed in two contrasting ways, leaving some professionals in a double bind. They felt if they enquired about “cultural” issues of which they were uncertain with patients or their families, their ignorance might be perceived as discriminatory by patients. Yet there was also concern that investing greater time or effort to address needs with some patients might be regarded as preferential treatment by other patients, or indeed professional colleagues (Box 2).

Relating their uncertainty, professionals usually situated perceptions of ethnic difference uppermost within the health care interaction. Other cultural influences such as diversity of social background, education, age or gender, or similarities across cultures, excited little discussion. However broader recognition of this arose on occasion (note: “P” designations refer to the interviewees in excerpts with more than one response; each excerpt is followed by the interviewees' professional category):

P3: *…People from different cultures, I believe, have a different disease model. Sometimes it's very difficult to tune into that.* P2: *But I think it's very difficult to say that it's to do with your cultural, your ethnic background. …I think I would find it as difficult to understand and empathize with the belief systems of the deeply religious Roman Catholic Geordie [the term “Geordie” refers to a person from Tyneside, UK] …as with a not particularly religious, middle-class Asian person who might happen to share my belief systems. So, I don't think you can necessarily equate cultural differences with ethnicity.* (Physicians, Group 14)

### Focus on Cultural Expertise

Respondents appeared to tackle their uncertainty by seeking or applying rules to guide their behaviour in cross-cultural encounters. They focused on their ignorance and need for factual knowledge about cultural differences in order to act and feel more confident. Some had been exposed to training to approach patients from ethnic minorities in this way. Others indicated they had little or no experience of relevant training but had assimilated this approach as part of received wisdom. Professionals accordingly emphasised improving their cultural expertise or those of others as the route to achieving “culturally appropriate” outcomes (Box 3).

Professionals' accounts exposed a dissonance in their work. There was incongruity between, on the one hand, their desire for and application of cultural expertise using a largely rule-bound, knowledge-based framework and, on the other, their continuing experience of uncertainty with patients despite this approach. Professionals identified where their varied knowledge of differing cultural issues had been helpful, for example insights into variation in cultural expectations or attitudes:

*I'll tell you one of the problems…tuning in to Kurdish people. If you don't you just finish up getting more and more frustrated and…and just one very small piece of knowledge about people's expectations or the way they relate…can actually cause the whole penny to drop I think…* (Physician, Group 14)

P3: *And attitudes. Like (X) said, they tend to come and want a doctor right away, they are not used to an appointment system which can come across wrong if you don't understand that.* (Primary care nurse, Group 13)

**Box 1. Professional Uncertainty**

P3: *I think you have to be very careful because the rule that we understand might be quite different. You know, if you go and touch somebody it could be completely inappropriate. We might feel that we are touching somebody to show them we empathize in our culture, but if we did that to an Asian gentleman it might be quite the wrong thing so I don't know… P4: I think I feel less confident in knowing what the expectations were. Like I feel fairly comfortable about breaking bad news to seriously ill people…from a background that is more common to me. I would actually find it much more difficult to say to an Asian family, where I'm not sure what kind of beliefs, expectations, what the family views are…* (Physicians, Group 14)

*What you learn, what you do with everybody is no different. But sometimes, it's, it's fear initially…that you're going to do something wrong.* (Palliative care multidisciplinary team, Group 9)

*I'm quite ignorant of the different cultures. I'd go in to a person of ethnic minority not as relaxed, generally more uptight… You're on edge…because you're thinking I don't want to put my foot in this, so that can make them uneasy with you.* (Occupational therapist, Group 12)

P1: *My inclination is to touch people, to hold their hand…but I wouldn't know if I had offended them because I don't know what culturally is allowed…* P2: *In a way, that makes us defensive; …you go into a situation thinking I've got to get it right. I mustn't say or do the wrong thing and that can affect your relationship with the person…* P1: *I don't really think we understand other cultures…We can always very easily put our opinions (that) we think this is what's happening … but we can't do that, because other people's cultures are different. We only sort of see the outside of what's actually happening within these cultures. I think that's where patients lose out really.* (Clinical nurse specialists, Group 1)

P9: *(The) Chinese don't have a word for cancer and its absolutely considered taboo to actually talk to somebody with cancer and say they've got it…so how do you start to talk to them about giving them chemotherapy if you're not actually allowed to use the word cancer…* P5: *(He) had a leg ulcer and it took a very, very long time for him to be prepared to roll his trouser leg up so that I could get near it. …And I found that very awkward…feeling terribly vulnerable because I didn't want to insult any of their cultural beliefs and yet at the same time I was trying to do a professional job for somebody.* (Palliative care specialist team, Group 8)

However, uncertainty remained where professionals found that stereotypical knowledge did not apply to a particular patient or family. While some participants suggested that they might best ask and explore the issue with the patient directly, their fears of causing affront or doing the wrong thing could stymie exploration (Box 1). Many professionals thus felt overwhelmed by the increasing ethnic variation of their patients. For example:

*Instead of thinking this is a patient, …treat them exactly as we do any other patient …you get overwhelmed with the fact that it's an ethnic group instead of a person.* (Palliative care team, Group 4)

Most respondents saw a need to learn about the perceived fundamental aspects of different cultures, seeking reassurance from guidelines on cultural difference. However, there was also some recognition of people's culture as more dynamic and multifaceted than is reflected in guidelines. Some noted variation within ethnic groups and across generations and the danger of patients becoming stereotyped, and highlighted the need to respond to people as individuals. For example:

P5: *Of course, we have got mixed up cultures now, haven't we? We have got second and third generations of children and grandchildren, fairly westernised in many families…quite hard to get our heads round isn't it? Because we don't quite know who we're dealing with…* P4: *Even though you might say this person is Polish, within that there will be all sorts of different likes and dislikes, preferences, cultural differences, everyone is different...* (Palliative care team, Group 4)

*It's not a production line you know. Every person is individual. Miss X's needs are different from Miss Y, whether they have the same disease. It's really not, you can't just say in black and white…It depends, person to person. Every case is different.* (Physician, Group 17)

There were few instances of professionals acknowledging their uncertainty openly with patients or exploring the patient's particular perspectives, concerns, or beliefs. Similarly, examples of positive learning or constructive outcomes from negotiating uncertainty were rare. There were, however, “deviant” cases [31], albeit in a context of a continuing challenge of cultural difference. For example:

P3: *To think about how you speak to people and to think about what the family and what the patient themselves most want to know and how they want to know it.* P5: *It's listening isn't it? It's being aware of, you can't just say this is a Muslim family therefore this is how I'll do this as a template… You have to be able to modify how you're going to deal with these situations…* (Physicians, Group 16)

*I think what's helped me, it's very much built into counsellor training…is a model around working with any kind of difference rather than around checklists…and yet I still struggle to know how to meet people who are very, very different to me.* (Multidisciplinary hospice team, Group 9)

**Box 2. Uncertainty about Equality and Equity**

P5: *Is it racist to ask these specific groups, is it racist to ask them because they're Asian, about eating habits, about these sort of details about washing or praying?* (Physiotherapist, Group 2)

P1: *If you want to be reflecting someone's culture you can be seen by colleagues as being over the top and not giving them, the same as everyone else, there is some inequality about it because you are making allowances for somebody's culture or ethnicity or whatever, that you are not being fair to everybody else.* P2: *We are doing something more for someone else than we are doing for the majority of the patients and I think that is a very dangerous ground to tread, that we have to be seen to be trying to make everybody equal and not doing more for someone else. I think we quite often achieve this, of bending over backwards to help other people, now I think that's right, that we should be doing that, but the honest answer is that some people don't think that's right.* (Clinical nurse specialist, Group 1)

### Professional Disempowerment

The uncertainty professionals experienced had disempowering effects. Professional disempowerment was characterised by anxiety and stress in cross-cultural interactions and inertia in their clinical approach.

Respondents worried about asking or were unsure about how to ask patients about their values, perspectives, or practices. Some respondents who had experience of diversity or cultural competence training continued to feel ill-equipped to respond to patients' needs. So pronounced was the fear of “getting it wrong” or affronting patients that avoidance of issues perceived as problematic or potentially emotive could occur. Professionals' ability to think and act flexibly or creatively seemed to falter. A disabling hesitancy and inertia in practice was the common consequence, compromising satisfactory interaction with patients. This hesitancy was further manifested in some respondents' perceptions, or those of their colleagues, that only professionals of similar ethnic background to the patient were best placed to offer appropriate care (Box 4).

Respondents' self-perceived low cultural expertise and uncertainty made them uncomfortable as professionals, and some felt that quality of care for patients was being affected as a result. This was further enmeshed with professionals' concerns about the lack of rapport they felt able to achieve with patients because of a perceived lack of shared cultural background, language, or values. They felt less able to use, or trust, empathy as part of their approach. Thus, for example, professionals wondered when it was acceptable to touch someone, what a fellow professional from the same culture as the patient might do, or whether they might do the wrong thing and compromise their relationship with the patient (Boxes 1, 2, and 4).

However, there were isolated examples of professionals feeling more empowered when cultural expertise received less emphasis:

*I attended a workshop…(the message was) we all have basically the same needs…don't get yourself tied up…because you're worried… We have to try to find a way of making sure that people can access services. For me, that was quite freeing really…I don't have to know everything about every religion, every culture...I found that very helpful and I think it could be easy to hide behind well I don't know and perhaps they need something different and I can't give it…* (Palliative care multidisciplinary team, Group 9)

### Uncertainty and Disempowerment as Self-Perpetuating

To illustrate how respondents' experiences and perceptions may be linked to effects, the categories of professional uncertainty and disempowerment that developed from our analysis are typified in Box 5 as common health professional perceptions and responses.

It is hypothesised that professionals' uncertainty and disempowerment have the potential to be self-perpetuating. Self-perpetuation may occur within the context of professionals foregrounding cultural difference, and in particular their desire for, and application of, a rule- bound, knowledge-based cultural expertise. This phenomenon may preclude opportunity for learning as the categories interact to limit new discovery and constructive experience with patient diversity, thus reducing learning for future encounters.

## Discussion

Our findings highlight the considerable uncertainty health professionals may experience working with patients of differing ethnicity to their own, alongside professionals' emphasis upon knowledge about cultural difference. This uncertainty may disempower professionals, creating hesitancy and inertia in their clinical practice to the potential detriment of patient care.

### Methodological Considerations

The findings must be interpreted with regard to the study context and sample. Participants were likely to have been particularly interested in ethnic diversity and health care. Their accounts showed significant awareness and understanding in this context, which may reflect this interest. In addition, although use of interaction in focus groups offers the advantages described, a recognised challenge is the possibility of group dynamics promoting uniformity of views [30]. We sought to address this potential limitation by emphasising our interest in differing perspectives as equally important and valid within groups, and by including a range of group compositions.

**Box 3. Focus on Knowledge-Based Cultural Expertise**

P2: *I mean if there was some kind of resource. If there was some brief thing that showed in this culture, these are important things to bear in mind about communication and these are the kind of things you need to be aware of when you see them. I think just something as simple as that would actually be quite helpful.* (Primary care physicians and nurses, Group 15)

P8: *You went away (from training) with a piece of paper that said quite specifically this religion, watch out for such and such, just be aware of it. It's something I still use and give to students…P6: I've recently written an article on what to do at the time of death…and the editor wrote back to me and said ‘could I include a table on what to do for different cultures?'* P2: *Sometimes a checklist stops people from feeling anxious… We often have people who say, ‘I just don't know what to say, what shall I call him?'* (Palliative care team, Group 9)

*What was right and wrong, some guidelines as to how we should behave and what is acceptable…we would go in much more at ease and then obviously our attitude would be better* (Occupational therapist, Group 12)

P3: *They [other health professionals] should go to…multicultural training awareness days…* P4: *I think a little knowledge about religion, background, values, tradition does help.* P7: *Some people are not culturally aware. My children wear topknots [of unshorn hair in males as Sikh article of faith]…most people call them girls. Some people don't even know the difference between cultures...some people are not aware at all.* (Bilingual link workers, Group 5)

While active inclusion of those willing to articulate their experience is key to qualitative enquiry, we recognise our participants' perceptions may not be typical of all health professionals or other settings. However, a broad range of health professionals with varying characteristics were included, data generation continued to saturation, negative cases were sought [31], and we undertook validation with participants [32] and other groups. These methods increase the qualitative rigour of the study and the likely relevance and transferability of the findings beyond the immediate study. Further research involving individual interviews with health professional respondents, observation of their practice, and exploration of the perspectives of patients, would be of value in extending the current data and understanding in this field. We are currently conducting a parallel study with patients in this context.

We recognize the potential influence of our own, largely health professional, backgrounds on data interpretation. However, we have attempted to lay emphasis upon what participants said and note they offered apparently candid reflections on their experiences and concerns, despite the possible sensitivity of the topic. While our findings may possibly have been more readily exposed within the emotive context of care relating to cancer, they seem unlikely to be unique to this setting.

### Uncertainty and Disempowerment Responding to Cultural Diversity

Uncertainty is well recognized in a range of health contexts, for example, in clinical decision-making in relation to evidence [33–35] or as a central theme of complexity in care [36]. This study is, to our knowledge, the first to identify professionals' more personally felt uncertainty and disempowerment working with patients of differing ethnicity to their own. The study invites the question of why these problems remain despite the promotion of “cultural competence” [19]. Their origin, given respondents' emphasis on their lack of cultural expertise, may partly reflect the dominance of expert knowledge as the traditionally valued attribute of the professional, formed in their training and culture as health professionals. Contemporary notions of cultural “competence” in health care [22–26] can imply a technical skill that can be mastered and circumscribed. Yet a “technical mastery” approach is unsuited to negotiating the uncertainty inevitable within intercultural encounters, given the kaleidoscopic, fluid nature of patients' cultural diversity.

The focus of cultural competence in relation to others can be contrasted with professionals' relative lack of attention to examining their own culture and that of biomedicine in particular. This field has its own set of interests, biases, and influences on care, awareness of which might inform cross-cultural care. Fadiman, for example, has highlighted the cultural gulf between the illness and treatment beliefs firmly held by parents of a gravely ill child and the equally uncompromising medical convictions of the American physicians who cared for her, with tragic results and no easy resolution [37].

Becoming more comfortable with cultural difference and the uncertainty it creates may require greater development of self-reflection on one's own cultural identities as an individual and as health professional. This process implies becoming more aware of the degree to which one's perceptions and behaviours are culturally conditioned. However, health professionals' inclination may be to resist such efforts because awareness is as much “emotional” and derived from experience and reflection as it is through the more traditionally valued intellectual process of handling information [38]. In addition, people prefer to see themselves as autonomous rather than as subject to cultural influences; anything that may question or challenge the nature of one's identity is threatening.

The consequences of professionals' uncertainty, hesitancy, and inertia in their practice are of serious concern. Patient perspectives and expectations may remain unexplored or critical issues unaddressed. This gap may leave care to be based upon inadequate information and professionals' cultural assumptions and generalization, which may compound unhelpful stereotypes and attitudes that some health professionals may hold [39,40]. Such difficulties may be further exacerbated by difficulties in communication as a result of language barriers or interpreting.

Professionals' uncertainty and disempowerment, and the potential of these two phenomena to perpetuate each other, may contribute to institutional racism [41] in health services. Our results add to debate about the possible contribution of health care providers to ethnic disparities in health care, in particular the hypothesis that their behaviour may be influenced by patient ethnicity [27]. Our results have parallels with evidence that physicians engage in longer consultations and volunteer more information with patients of similar middle class backgrounds to themselves, compared to patients from less well-educated, lower social class backgrounds [42–44].

With greater awareness of discrimination in services [41], it is perhaps unsurprising that professionals worried about causing affront, being “culturally inappropriate,” or being discriminatory and that they sought certainty in guidelines to inform their practice. At the same time professionals in this study were uncomfortable with their uncertainty and recognized that they may be failing patients. Given that our sample may have been particularly interested to reflect on their practice, the challenges experienced by other professionals may be even greater, or perhaps lie unconsidered.

**Box 4. Professional Disempowerment**

P1: *I suppose there are differences in the culture you are brought up with, they are mostly intuitive…I'm not sure I have the same feeling, or certainly in my everyday experience of dealing with patients from minority groups… I haven't got the same feeling of trusting my kind of sense of what's going on because when can you put your arm round a patient?* P2: *Yeah, that kind of thing.* P3: *Yeah. Which you do intuitively, don't you?* P1: *And therefore there's the danger of doing it inappropriately. P3: Yeah. P1: You don't know which way to go, so you don't necessarily kind of progress.* (Primary care physicians and nurses, Group 15)

*Everybody was so upset (Asian man dying from liver cancer) it was very difficult to ask questions about cultural things…we did have some diversity training but it was so broad that it wasn't very personal to the situation really…I just felt very inadequate.* (Occupational therapist, G12)

P1: *You could frighten yourself though couldn't you? You could stop yourself doing so much with people, being so frightened to offend or do something wrong.* P2: *And that's what builds the barrier, isn't it? Sometimes we are a bit, too careful, because we don't know what's right or wrong.* (Clinical nurse specialists, Group 1)

*I said…a greeting in Muslim, but they were Hindu, but I didn't know because from outside I really couldn't tell. And when I went in and I saw the pictures and I thought, ‘Oh my god', I didn't know, and I'd said it at the door…and then I went in and I just shut up.* (South Asian link worker, Group 5)

*…it just makes you feel so helpless, doesn't it. You wonder…if [another health professional] from the same culture had turned up that day, how would she have dealt with it? Would she have, you know, have gone to the patient or would she have gone through the family? How differently would she have acted?* (Palliative care team, Group 4)

*I think inherently some people really, really struggle with caring for people who are not from the same ethnic background as themselves…I can see it in some team members…this assumption that black nurses will look after the black patient, it's there.* (Physiotherapists, Group 2)

### Opportunities and Implications for Practice

The findings might inform interventions to enhance quality of care, and also professionals' experience of working with diversity. First, uncertainty should be acknowledged and legitimized as inherent to negotiating care responsive to patient needs. Professionals might be supported to reflect on their responses to uncertainty [28] and its potential to create inertia in their practice. This process might accompany other learning to handle uncertainty as central to other aspects of clinical care [33–35].

Second, a shift in emphasis away from knowledge-based cultural expertise toward a greater focus on the patient as an individual is needed. Perhaps unsurprisingly, defining “cultural competence” adequately for training and practice has continued to prove elusive, with significant evidence of its effectiveness yet to emerge. This study suggests the promotion of “cultural competence” as simply reducible to a set of technical knowledge and skills may have the paradoxical effect of increasing professionals' uncertainty and reducing their confidence responding to cultural diversity.

Classic ethnographies of health care demonstrate that to be sensitive to cultural difference is more than a question of knowledge [45,46]. Rather it requires a more human and emotional investment to connect successfully with those whose world differs from one's own. Such ethnographic methods place central importance on understanding another's point of view, values, and practices, by empathising with their experience and engaging with their differences or “foreignness” [47,48]. In the light of the current research this approach offers a way forward for health professional practice because it becomes possible to actively embrace, rather than be uncomfortable with, the uncertainty that cultural diversity creates.

Third, it should nevertheless be recognised that, as demonstrated here, health professionals and those in training [17] do sense an understandable need for more content-based information about cultural and ethnic differences, and it remains important to address this need in training. Discussion of culturally specific issues and examples can engage health professionals, naturally interested in “difference,” and provide a practical context for learning [15,17]. However, this engagement must be balanced with avoidance of stereotypical assumptions and application of transferable principles flexibly for any health encounter, thus enabling learning for new and unfamiliar situations [15,17,49].

Fundamentally, health care professionals need to be supported to respond to patients as individuals [49], whose cultural diversity embraces not only ethnicity, but other influences such as gender, social background, and education. They should be able to reflect on their own cultural influences as health professionals and have the confidence to explore patient perspectives where there is uncertainty. Following an ethnographic approach, this requires asking individual patients about their views and what matters most to them in their experience of illness and treatment, rather than assuming cultural knowledge of the patient [50]. This approach may empower professionals to use individuated patient information to facilitate assessment of needs and understanding of expectations to enhance care.

**Box 5. Ethnically Diverse Patients: Health Professional Perceptions and Responses**

**Health Professional Perception (Category of Uncertainty)**

People from other cultures are different from me.

If I ask a patient about their cultural needs I might offend them or they might think I am racist.

If I don't ask the patient about their cultural needs they might think I'm racist.

I am too ignorant of different cultures to be culturally appropriate.

Even with more cultural expertise I can never give the same care to a patient as a professional from the patient's own cultural background.

If I spend more time with a patient of ‘minority ethnic background' this may be perceived as favouritism.

Everyone else knows what to do.

**Health Professional Response** **(Category of Disempowerment)**

So I need to focus on the differences.

So I'd better not ask.

So I'd better ask, but how?

So I need more cultural expertise (or someone tell me what the facts are and what to do).

So it would be better if another professional of the same cultural background could step in.

So I'd better deal with everyone in the same way.

So I'd better keep my uncertainty to myself.

Professionals might be reassured that it is neither feasible nor desirable to be familiar with all aspects of the different cultural issues that may apply in an encounter. Rather they should recognize the importance of cultural issues and that exploring them where necessary, to understand patients' perspectives and empathize with their experience, may facilitate effective care. Developing this ability in the context of cultural diversity might also facilitate health professionals' greater engagement with the more social, emotional, and humanist components of health care practice [51].

Fourth, facilitating greater awareness of concepts of equity—that is, of being fair as distinct from treating people equally—may help those professionals with dilemmas about equality to understand the appropriateness of varying responses to patients to meet similar care needs. Differing investment in time, enquiry, and possibly greater requirement to negotiate care, may be needed to achieve equitable outcomes.

Such strategies may help professionals to work constructively with diversity and uncertainty. They may also reduce the sense of professional compromise some respondents felt. Any steps must be integrated with other efforts to support intercultural communication and reduce misunderstandings caused by differences in language and communication style [52–54]. Further, the question of why previous learning in this field may have had only modest penetration into thinking and practice merits reflection. That from the social sciences, for example [45,46], is often neither readily available within the biomedical literature nor accessibly presented in less esoteric form. It might also be argued that social science's demonstration of the social and cultural character of medicine forces a questioning of the privileged status of biomedical knowledge. More might be done to facilitate better access to such learning. Further applied research in the field, including assessing the effectiveness of culturally informed practice in health care, is also much needed.

The challenges of wrestling with diversity have been little presented from health professional perspectives. The current study has sought to contribute by providing empirical data that may speak more directly to health professionals. Identification with the uncertainty expressed might offer a first step to acknowledging its existence and to its negotiation in practice. The extent to which quality and equity of care may be enhanced by interventions informed by this study are hypotheses for future testing. However, failure to empower health professionals to accept and work creatively with uncertainty may have significant potential to perpetuate inequality in the experience of health and health care of our ethnically diverse populations.
